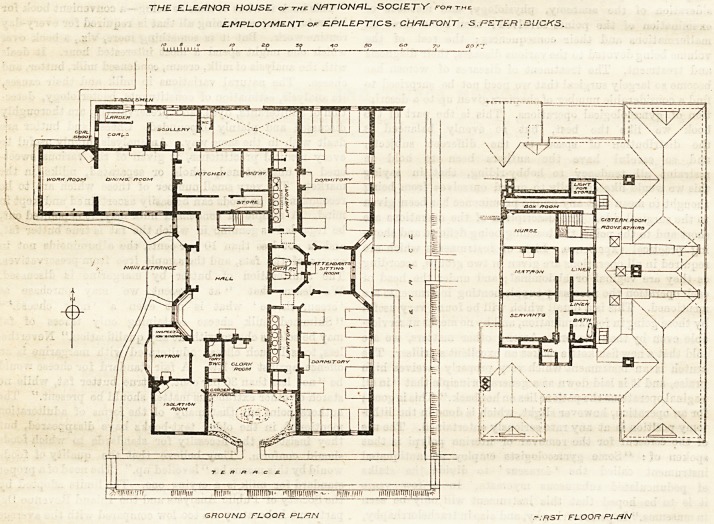# Hospital Construction

**Published:** 1897-12-25

**Authors:** 


### The Institutional Workshop.

### HOSPITAL CONSTRUCTION.

### HOMES FOR EPILEPTICS, CHALFONT ST. PETER.

The Home for Women shown on the plans published herewith, has been erected by the National Society for the Employment of Epileptics, at the expense of Mr. Passmore Edwards, who is also erecting for the same Society the adjoining Home for Men. The Women's Home, designed by Mr. E. C. Shearman, is a picturesque building, containing accommodation for 24 "colonists" as they are called, in two large dormitories of 12 beds each (with an attendant's room between them), a hall with western aspect, serving as a day-room, and a dining-room and work-room facing south. The attendant's and matron's rooms are planned so as to give the requisite supervision over the inmates, and an isolation-room is provided in the south-west corner.

The arrangement of the various rooms and offices is very different from what is commonly seen in hospitals. It is held, however, that this is not a hospital but a dwelling-house, and a careful inspection of the plans will show that many of the special features by which they are characterised have been introduced with the object of facilitating the supervision of the inmates by as small a staff as possible. The position of the kitchen, close both to the hall and the dining-room; of the matron's room, adjoining the hall on the one side, and the isolation or sick room on the other; and the position of the attendant's room, between the two dormitories and close to the lavatories and bathroom, are thus to be explained; and this probably has been the object of the peculiar position of the sanitary conveniences. These are placed between the main buildings, the aim, doubtless, being to make them accessible both from the day-room and the dormitories. The same in regard to the lavatories. These are intended to serve also as dressing-rooms, and are therefore provided with lockers for the clothes of the inmates, an arrangement which will no doubt conduce greatly to the sweetness of the dormitories. The separation of the air of these conveniences from that of the living and sleeping rooms is to be provided for by lantern ventilators placed over the lavatories themselves and the connecting lobbies. How far this arrangement will turn out to be successful is a matter which we shall watch with considerable interest. The "cut off" appears to depend on the freedom of ventilation which is maintained in the lobbies, and as these lobbies form the only means of communication between the hall, or living-room and the bedrooms and the attendant's sitting-room, we have our doubts.

The accommodation is planned chiefly on one floor, in view of the condition of its inmates, but there is a small upper storey containing the matron's and attendant's bedrooms, with a bathroom and closet.

THE ELEANOR HOUSE OF THE NATIONAL SOCIETY FOR THE EMPLOYMENT OF EPILEPTICS. CHALFONT, S. PETER. BUCKS.

GROUND FLOOR PLAN FIRST FLOOR PLAN

The Men's Home, designed by Mr. Maurice B. Adams, has the same accommodation as the other, but is planned on more usual lines, a corridor connecting the various rooms for inmates and terminating at the kitchen, placed at one end of the building, its small size being due to the existence of a separate administrative block, from which all the principal meals are served. The inmates' closets, however, seem to have no windows or through ventilation, such light and air as they receive being obtained from the opposite side of the room, containing the lavatory, in which they are placed. The isolation-room in this home is on the upper floor, where are also the matron's and servants' rooms, a closet, and a top-lighted bath-room.

While wishing every success to the Society in the work which it has undertaken, it seems desirable to point out that the plans of its most recent buildings do not appear to come up to what we may term hospital standard, and although it may perhaps be fairly maintained that these are not hospitals, it must also be maintained that institutions intended to be occupied by 24 people and upwards are not ordinary dwellings. We look upon these homes for epileptics as having a scope of usefulness extending far beyond the mere question of providing their inmates with employment. The problem they ought to help to solve is, how far the treatment of these cases by healthy occupation, simple food, and healthy surroundings will succeed in stopping the manifestations of the disease, and for this purpose we think that the health conditions should conform rather to the standard of the hospital than to that of the dwelling house.

## Figures and Tables

**Figure f1:**